# Effects of Technical Textiles and Synthetic Nanofibers on Environmental Pollution

**DOI:** 10.3390/polym13010155

**Published:** 2021-01-03

**Authors:** Ali Aldalbahi, Mehrez E. El-Naggar, Mohamed H. El-Newehy, Mostafizur Rahaman, Mohammad Rafe Hatshan, Tawfik A. Khattab

**Affiliations:** 1 Department of Chemistry, College of Science, King Saud University, Riyadh 11451, Saudi Arabia; melnewehy@ksu.edu.sa (M.H.E.-N.); mrahaman@ksu.edu.sa (M.R.); mhatshan@ksu.edu.sa (M.R.H.); 2 Textile Industries Research Division, National Research Centre, Giza 12622, Egypt; mehrez_chem@yahoo.com (M.E.E.-N.); tkhattab@kent.edu (T.A.K.)

**Keywords:** technical textiles, synthetic fibers, nanofibers, natural fibers, finishing, environmental protection

## Abstract

Textile manufacturing has been one of the highest polluting industrial sectors. It represents about one-fifth of worldwide industrial water pollution. It uses a huge number of chemicals, numerous of which are carcinogenic. The textile industry releases many harmful chemicals, such as heavy metals and formaldehyde, into water streams and soil, as well as toxic gases such as suspended particulate matter and sulphur dioxide to air. These hazardous wastes, may cause diseases and severe problems to human health such as respiratory and heart diseases. Pollution caused by the worldwide textile manufacturing units results in unimaginable harm, such as textile polymers, auxiliaries and dyes, to the environment. This review presents a systematic and comprehensive survey of all recently produced high-performance textiles; and will therefore assist a deeper understanding of technical textiles providing a bridge between manufacturer and end-user. Moreover, the achievements in advanced applications of textile material will be extensively studied. Many classes of technical textiles were proved in a variety of applications of different fields. The introductory material- and process-correlated identifications regarding raw materials and their transformation into yarns, fibers and fabrics followed by dyeing, printing, finishing of technical textiles and their further processing will be explored. Thus, the environmental impacts of technical textiles on soil, air and water are discussed.

## 1. Introduction

A technical textile can be defined as a textile material and product manufactured mainly for its technical and performance characteristics rather than their artistic or ornamental features [1,2,3,4]. Such a short definition obviously affords a significant scope for an explanation, particularly when an increasing amount of textile-based merchandise is merging both technical performance and *aesthetic* characteristics and acts in an equivalent measure. Technical textiles have been utilized for a variety of applications, including automotive purposes such as safety belts, medical field such as surgical sutures, geotextiles such as separation fabrics for soil layers, agriculture such as horticulture fabrics for protection from solar radiation, and protective textiles such as superhydrophobic fibers. It is a huge and increasing segment that supports a huge group of other industries [5,6,7,8,9,10]. Currently, technical textile materials are mainly used in filter garments, furnishings, medical hygiene and construction materials. The global market for high-performance textiles is rising as never before. The market size of high-performance textiles has been projected to surpass about US$251.82 billion by 2027. It was estimated at US$175.73 billion in 2019 [11]. High-performance textiles will have a similar story all over the world, being very strong, durable and versatile. With increasing demand and consumption, the difficulty of dumping will also increase. Among the most demanding users of high-performance textiles are armed forces, while army workers are among those with the serious requirements. Technical textiles have demonstrated to be the main supplier to all these defense purposes replacing the traditional heavier merchandise [12,13,14,15,16,17,18]. There are major differences between technical textiles and conventional textiles industries [12,13,14,15,16,17,18,19]:

(a) Technical textiles are favored for their extremely precise performance quality, and consequently they are more expensive than conventional merchandise.
(b) Technical textile producers must use accepted testing techniques in order to gain customers’ trust concerning standard specifications.
(c) Technical textiles are for a certain sector of a market that requires more flexible production schedules and smaller manufacture spells.
(d) Technical textiles producers usually have to be prepared to spend on research and development [19].

**Figure 1:**
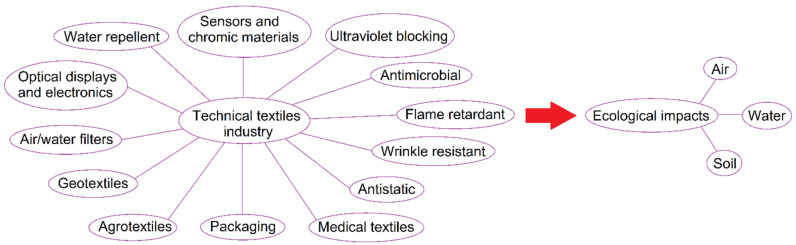
Schematic diagram representing various applications of technical textiles, including protective, medical, packaging, agricultural and geological textiles, and their environmental impacts on air, water and soil.

The geometric facial appearance of the fibers facilitates the design, preparation of planar fabrics via weaving and knitting processes. These textiles are extremely porous, thus controlling thermal isolation, wind resistance, and vapor permeability such as sweat. In addition, textiles fibers have to be able to afford specific mechanical properties defined by particular values of fiber stiffness and elasticity which allows for fabric structure deformation, and incorporation of colorants. Transport technical textiles are involved in airplanes, trains, automobiles. and boats, which depend strongly on high-scale technical parts possessing a very low weight while demonstrating concurrently a high stiffness and strength [20,21,22]. The exploration for an all-embracing term describing such non-conventional clothing is not limited to ‘Technical’ and ‘Industrial’. Terms, such as “High-Performance Textiles, Functional or Smart Textiles, Engineered Textiles and High-Tech Textiles” have been employed in different perspectives. However, the term “High-Performance Textiles” is often used to describe the activity of textiles [23,24]. This critical review article presents a systematic survey on the development of technical textiles providing a bridge between manufacturer and end-user. The accomplishments in advanced applications of technical textile material have been proved in different fields. Raw materials and their transformations into yarns, fibers and fabrics; followed by dyeing, printing, finishing and their further processing are explored. The environmental impacts of technical textiles on soil, air and water are discussed. The techniques and solutions allied to reduce those ecological impacts from technical textile industry were also explored. The future of high-performance textiles promises even more stronger worldwide competition seeking more applications, high-quality, lower cost and environmentally-friendly products. As shown in Figure 1, we discussed the advanced applications of high-performance textiles as a bridge between manufacturer and end-user, the sustainability and environmental impacts from those textile products, and methods applied to reduce the ecological harmful effects generated.

## 2. Types of Fibres for Technical Textiles

**Figure 2:**
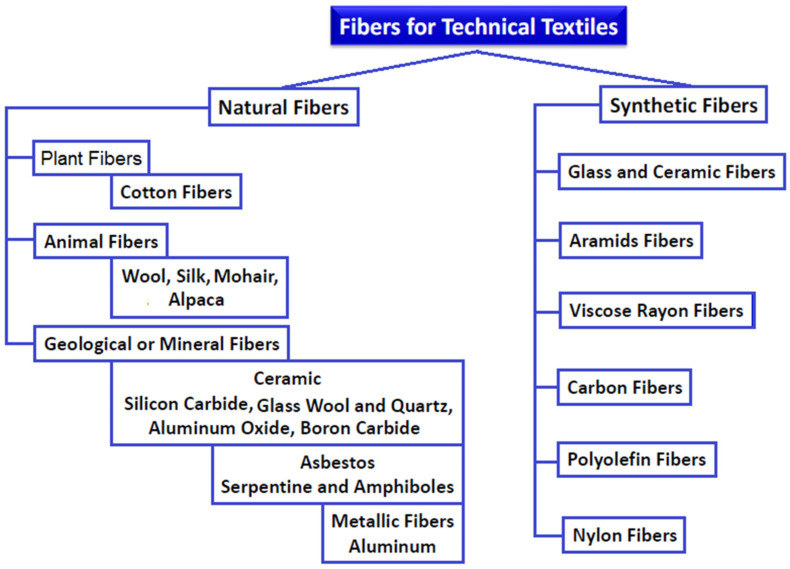
General classification of fiber-based technical textiles into synthetic and natural fibers.

There are a variety of yarns have been applied for high-performance textiles, including natural as well as man-made yarns depending on the end product. There are different structures of yarns, such as staple, monofilament, multifilament, texture and twist, which are produced by different spinning manufacturing techniques, such as friction, ring, air-jet and rotor [19]. Special characteristics can be obtained by different yarns to afford particular functional requirements of technical textiles according to the end-use application, such as packaging, medical agriculture, protective, filtration and geotextiles. Natural fibers are characterized by high modulus/strength and moisture intake as well as low elasticity and elongation. Regenerated cellulosic fibers possess low modulus/strength and elasticity as well as high elongation and moisture intake. Synthetic fibers, such as nylon, polypropylene and polyester, possess high modulus/strength and elongation with an acceptable elasticity and comparatively low moisture intake. Natural fibers can be divided into plant, animal and geological origin. Plant fibers possess excellent engineering characteristics, while animal fibers possess a lower modulus/strength as well as higher elongation than plant fibers. Geological fibers are costly, brittle, and lacking strength and flexibility. Figure 2 displays a tracking chart demonstrating a general classification of natural synthetic fibers [25].

### 2.1. Synthetic Fibers

**Figure 3:**
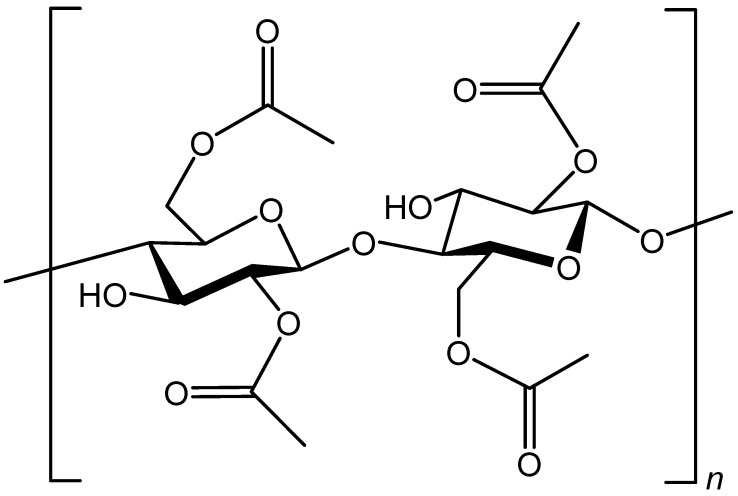
Chemical structure of cellulose acetate.

Synthetic fibers are man-made fibers developed to improve the properties of natural fibers. Nonetheless, not all man-made fibers are synthetic fibers. For instance, acrylic, aramids, dyneema, rayon artificial silk, vinyon, vinalon, acrylonitrile rubber, polybenzimidazole, nylon, polyesterzylon, glass, polylactic acid, metallic and derclon are synthetic fibers. On the one hand, cellulose acetate and rayon are known as regenerated or man-made fibers but cannot be considered as synthetic fibers. Synthetic fibers are obtained via an extrusion process of polymers prepared by reacting certain monomers through a process called polymerization. On the other hand, regenerated/man-made fibers are reproduced synthetic fibers from a dissolved natural material to result in fibers with different properties, such as regeneration of viscose from cellulose. Figure 3 displays the chemical structure of cellulose acetate [26].

Synthetic fibers can be customized for certain end-use applications by tuning their properties, such as length, decitex, tenacity, softness, stain resistant, fire/water repellent effects, surface profile, wrinkle free, finish and even by blending into hybrid product systems [26].

#### 2.1.1. Aramid Fibers

**Figure 4:**
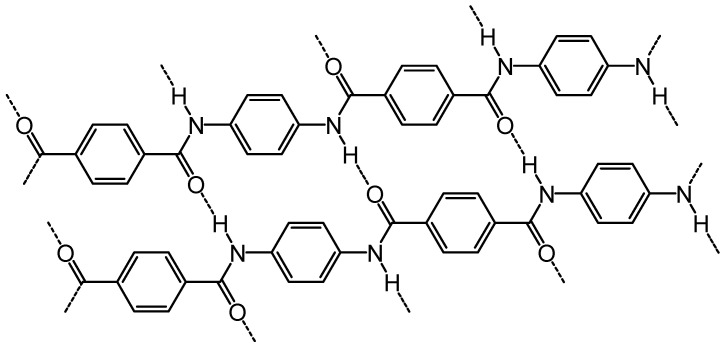
Structure of Kevlar, a *para*-aramid.

There are two types of amid fiber including the heat-resistant meta-aramids that are broadly used in heat protecting garments; and the other type is the high-strength and modulus para-aramids (Figure 4) employed in bullet resistant, tire reinforcement, hoses, friction materials, and ropes. The early victory of aramids was recognized in the development of carbon fibers, which have been accessible in the market since 1960s but mainly restrained by their high processing and material expenses to chosen highly valuable market, wind generator turbine blades, especially for aerospace purposes, sporting goods, and fuel tanks. In late 1980s, the introduction of other technical textile fibers was greatly increased for heat-resistant and flame-retardant uses from polybenzimidazole, ballistic protection and rope production, and ultra-strong high modulus from polyethylene, chemical stability from polytetrafluoroethylene, and filters from polyphenylene sulphide [27,28].

#### 2.1.2. Glass and Ceramic Fibers

Glass has been used as an inexpensive insulator as well as reinforcement of comparatively low performance plastics. It is now broadly used for various high-performance glass-reinforced composite purposes such as sealing, rubber reinforcement, filters, Pro-Tech clothing, packaging, and automotive industry to replace metal body parts and components. A variety of technical ceramic fibers have been developed; however, they are limited to comparatively specific applications owing to their high cost and poor mechanical properties [29,30].

#### 2.1.3. Carbon Fibers

Carbon fibers are generally manufactured from precursor fibers, such as acrylic which can be converted into carbon via a three stages heating procedure, including initial oxidative stabilization at 200–300 °C, followed by the carbonization stage by heating at 1000 °C in an inert atmosphere. As a result, both hydrogen and nitrogen atoms are expelled from the oxidized fibers, producing hexagonal rings of carbon atoms organized in oriented fibrils. The last step of the procedure is graphitization, occurring by heating the carbonized filaments at 3000 °C in an inert environment to raise the orderly arrangements of carbon atoms, which are arranged into a crystalline construction of layers oriented in the direction of the fiber axis, which is a significant feature in affording high-modulus fibers. Carbon fibers have been used in protective operations against skin irritation, and protection of processing equipment, auxiliary electric and electronic devices [31,32,33].

#### 2.1.4. Viscose Rayon Fibers

Viscose rayon synthetic fibers were first developed around 1910 and by the 1920s had significance in tires and other mechanical rubber products, such as conveyors, safety belts and hoses. Additional characteristics of viscose, such as heat resistance, high absorbance and appropriateness for processing by paper industry of wet laying methods added to its function as one of the original fibers employed for non-woven processing, particularly in disposable clean and hygienic applications [34].

#### 2.1.5. Nylon Fibers

**Figure 5:**
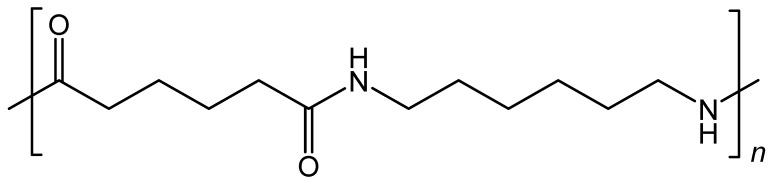
Structure of Nylon 6,6; a polyamide polymer derived from the condensation reaction of monomers-containing terminal amine (-NH_2_) and carboxylic acid (-COOH) groups.

Polyamides (Figure 5) first launched in 1939, affording high-quality elasticity and uniformity, abrasion and moisture resistance, and high strength. Its outstanding energy absorption is highly valuable in a variety of applications such as parachute garments, spinnaker sails and climbing ropes. Nylon-reinforced tires are still widely employed in developed countries, where the infrastructure quality of road surfaces is poor. In contrast to advanced countries, where regular road speed is higher and the heat resistance of viscose fibers are highly appreciated [35].

#### 2.1.6. Polyolefin Fibers

In the 1960s, the development of polyolefinic fibers such as polypropylenes and polyethylenes afforded cheap and simple processable fibers characterized by low density, high-quality abrasion, and moisture resistance properties, for a variety of applications such as packaging, carpet backing, and furniture linings. On the other hand, polyolefins are characterized by poor heat resistance and full water-repellency that have been turned into a benefit in nonwoven. Firstly, polypropylene employed in combination with viscose to allow thermal bonding for hygienic covers for diapers. Finally, the comparatively low extrusion temperature of polyolefins has been verified typically appropriate for the rapidly rising technology of spin laying [36].

### 2.2. Natural Fibers

**Figure 6:**
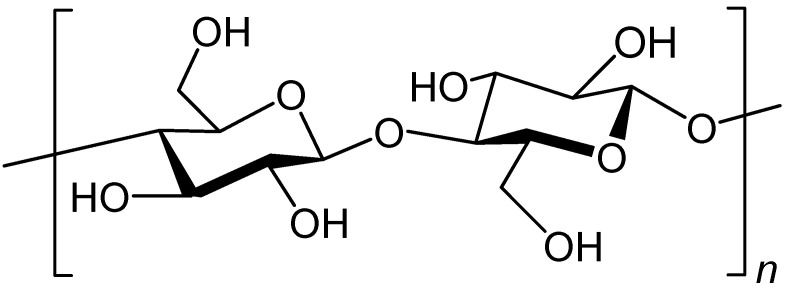
Chemical structure of cellulose demonstrating a linear polymer structure of D-glucose units.

Natural fibers are divided into plant, animal, and geological origin. Plant fibers (Figure 6), such as cotton, are the cell wall in both stem and leaf elements and consist mainly of cellulose, hemicelluloses and lignin [37].

**Figure 7:**
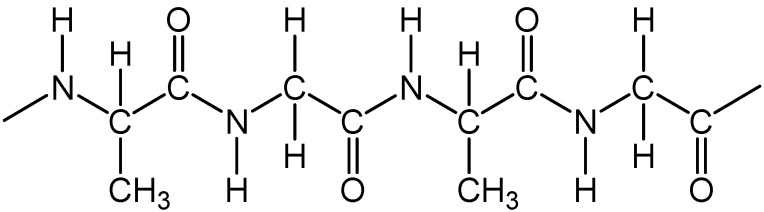
Silk chemical structure.

Animal fibers (Figure 7), such as silk, wool, mohair and alpaca. They are generally comprised of proteins, such as collagen, keratin and fibroin [25].

**Figure 8:**
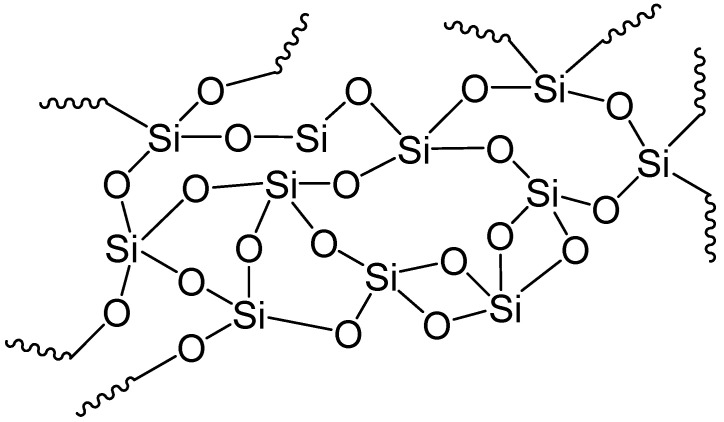
Glass fibers.

Geological or mineral fibers (Figure 8) are obtained from mineral sources. They can be employed in their original natural form or after a small amendment. Mineral fibers can be metallic such as aluminum; asbestos such as serpentine and amphiboles; or ceramic such as glass wool and quartz, silicon carbide, aluminum oxide, and boron carbide [38].

The most widespread textile fibers existing in the market of technical textiles are cotton and a number of coarser plant fibers, such as jute, flax and sisal. Due to their good tensile strength and stiffness, some natural fibers have been employed in technical textiles for different purposes, such as packaging, automotive, aerospace and fiber-reinforced composites [33].

#### 2.2.1. Jute Yarns

Jute is classified as natural multifilament fibers, which are characterized by durable, strong and easy to manufacture/dispose. Jute yarns are biodegradable and appropriate for a variety of weave densities. Woven jute textiles were originally used as geotextiles to prevent land sliding (deforestation), control of soil erosion by revegetation, and in jute-sand-mat constructions. Jute yarns have been employed for producing sacks of flexible packaging. Its characteristic physical properties have opened up novel opportunities for a variety of applications promoted mainly as a result of worldwide environmental concerns [39].

#### 2.2.2. Flax Yarns

Flax fibers are originally derived from the bast or skin of the stem of the flax plant. Flax fibers are characterized by softness, lustrous, anti-static, dries quickly and flexibility; bundles of fibers possess the look of blonde hair. Flax fiber is hollow and capable to absorb water up to 12% of its weight. Flax fibers are two-fold stronger than those of cotton and five-fold stronger than those of wool. Its strength raises extra 20% upon wetting. The longer flax fibers have been used in geotextiles. New applications for shorter fibers exist, such as packaging, automotive industry, asbestos replacement, panel boards, insulation and reinforcements for plastics and concrete. Flax yarns have been considered as environmentally friendly yarns with the ability to replace glass fibers in engineering composites [40].

#### 2.2.3. Coir Yarns or Rope

Coir yarns have been used as natural insulation materials obtained from flax fibers. Coir geotextiles cab be employed in soil conservation, erosion control and other civil and bioengineering purposes. It has the proper strength and toughness to guard land slopes against erosion whilst allowing plants to prosper. They can absorb extra solar energy and dissipate the energy of flowing water. The combination of both coir and flax yarns in woven form can be used for a variety of applications, such as binding purposes and to create matting. Coir yarns afford a reasonable solution to the problem of soil erosion and land sliding on the artificial slopes, such as motorways. 

### 2.3. High-Performance Nanofibers

**Figure 9:**
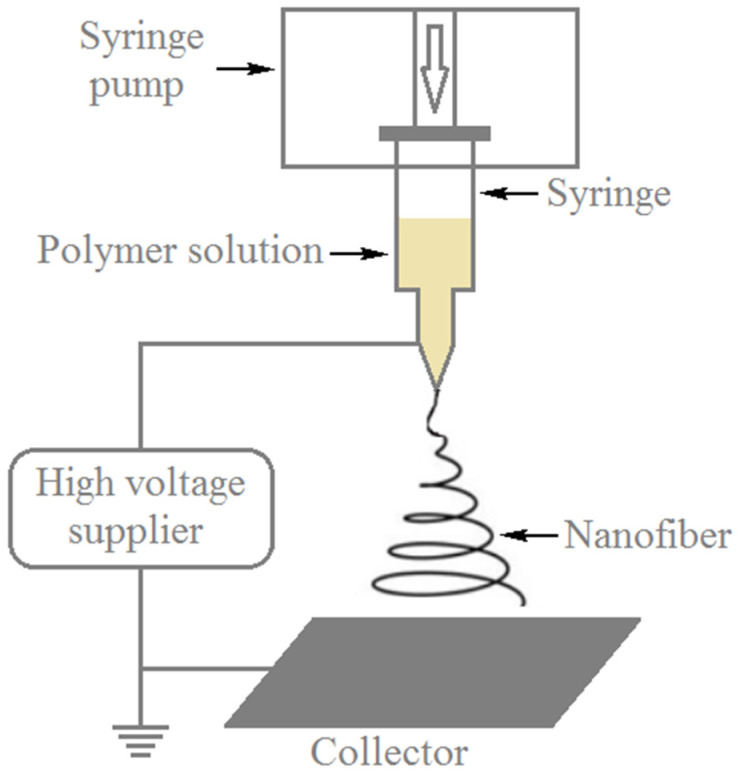
Diagram representing electrospinning apparatus.

Electrospinning allows manufacturing of nanofibers (Figure 9) mainly from polymer materials of both synthetic and natural origins [41]. The prosperity of different electrospun nanofibrous and non-woven architectures is exceedingly wide and it has been clear that highly fine fibers with diameters as low as few nanometers can be produced via electrospinning. Thus, such electrospun nanofibers and non-woven structures have been considered as major elements and systems, respectively, of nanomaterials. The electrons of inorganic molecules are usually delocalized and extended all over the bulk material, which can be considered as an advantage of the ability to control chemical and/or physical adjustments of size and geometry. On the other hand, organic-based materials show mainly localized electronic states of a molecular group, such as a chromophore. This results in that the electronic status is not influenced as the dimensions of the element, such as a fiber element is decreased to the nanoscale structure [42,43,44]. Electrospinning has been a facile and inexpensive method for the production of continuous nanofibers with large surface area and high porosity from a variety of materials, such as polymers, inorganic materials as well as inorganic/organic hybrids. However, the restricted control of pore size has been a major disadvantage of the electrospinning technology because the pore size affects the fiber diameter. Electrospun fibers have been applied in a diversity of applications, such as environmental remediation [45], filtration systems [46], protective clothing [47], sensors and biosensors [48], tissue-engineering scaffolds [49] and solar cells [50]. 

**Figure 10:**
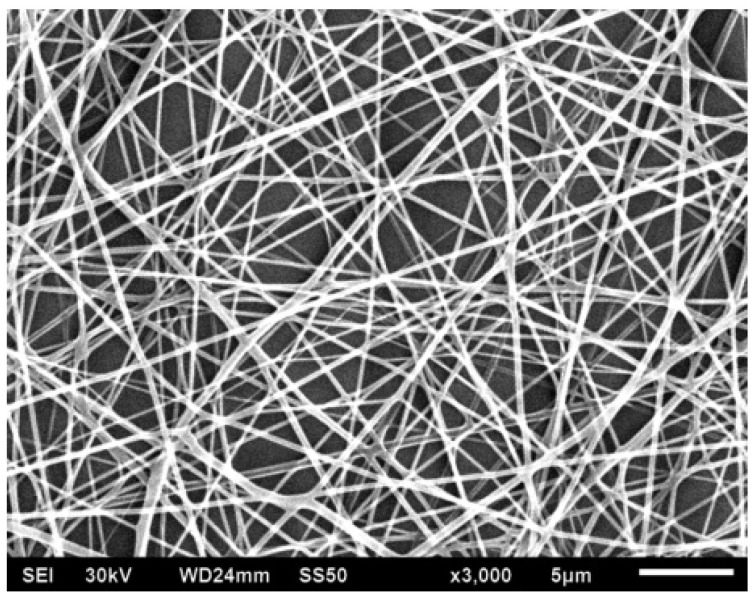
Scanning electron microscope (SEM) image of electrospun nanofibers from the polyacrylic acid in combination with a hydrazone chromophore [51]. “Reprinted with permission from John Wiley and Sons [51]”.

Nonwovens nanofibrous materials (Figure 10) can be employed to adjust the properties of traditional textiles of very thick fibers intended for clothing, furniture, hospital, and technical applications such as protective clothing from wind, self-cleaning, low temperature, and microbes [51]. This can be accomplished very efficiently, amongst other methods, by the deposition of nanofibrous thin layers of such non-woven nanofibers on the garments. Wind-resistant technical textiles depend on the average diameter of the pores within the nanofibrous thin layers on the surface of the fabric. The discovery is that the wind resistance of a fabric increases by five-fold of value as the average diameter of the pores decreased from 100 to 1 µm. This can be accomplished by replacing traditional fibers with pore diameter of around 10 µm by electrospun nanofibers with pores diameters of 100 nm. To accomplish such a high-quality wind resistance only a little coating degree around 1 g/m^2^ is required [52,53].

Positive features of such thin-layer deposited nonwoven nanofibers are that both resistance of humidity and vapor permeability are not affected. On the other hand, the thermal insulation of textiles increases by applying thin layers’ deposition of the nonwoven nanofibrous. The gas diffusion becomes limited as the pores’ average diameter is lower than the length of the mean free pathway of the gas molecules as a result of the domination of particle–pore-wall collisions, where the particle nanofibrous collisions and thermal conductivity is greatly decreased for nonwoven electrospun nanofibrous. It is clear that the thermal conductivity is decreased by numerous folds of magnitude as the diameter of the pores gets lower from 100 to 1 nm [54,55]. Coating fabrics by antimicrobial nanofibers is of considerable importance as a result of the high surface area of nonwoven electrospun nanofibrous that in turn leads to higher antimicrobial effectiveness. Antimicrobial active materials, such as traditional low molecular weight antimicrobial agents, metal oxides nanoparticles or oligomer/polymer ammonium materials can be integrated into the electrospinning composite to afford antimicrobial nonwoven nanofibers to be coated onto the fabric surface. However, such antimicrobial nonwoven electrospun nanofibers reflect some disadvantages, such as durability, adhesion on surface and releasing of the antimicrobial active agents that are hard to control. Self-cleanable textiles by superhydrophobic or photocatalytic nonwoven electrospun nanofibers can be produced *via* coaxial electrospun cellulose acetate with dispersed nanocrystalline titanium dioxide [56,57].

## 3. Functional Finishing of Technical Textiles

The term textile finishing includes a very wide range of activities performed on fabrics before commercialization. For instance, bed sheets are temporary hard-pressed before packing, and tints are treated by Pyrovatex to produce durable flame-retardant tenting garments, which have been significant to improving safety. Nonetheless, all finishing processes are considered to raise both attractiveness and function of the textile merchandise, such as water-repellency, which enhances the in-service performance of tenting garments. Further targets of textiles finishing may be described as an enhancement for purchaser satisfaction. This enhancement in the apparent value of a merchandise affords the base of up-to-date thoughts on the marketing of manufactured goods. High-performance textiles can be defined as textile products manufactured for non-aesthetic purposes, where their targeted function is the major criterion [58].

### Finishing Processes

The finishing processes can be divided into three major classes described as follows:

- Mechanical processing: This includes the passing of the material throughout machinery systems whose mechanical function usually accompanied by heating process, accomplishes the desired effects [58].
- Calendaring: Calendaring is accomplished by squeezing the fabric among two heavy heated rotating rolls to provide a modified fabric with a flattened smooth surface. The surface of the rolls is either smooth or engraved to afford the suitable required finishing to the textile material, whilst the manufacture of the rolls could be diversified from hardened steel to thermoplastic elastic rolls [59].
- Raising: Raising is a finishing method in which the surface effects can be produced to provide a brushed or napped fabric. It can be accomplished by teasing out the single fiber from the yarn using a teasel which was nailed to a wooden panel and the fabric was drawn over them to create a cloth with a hairy surface, which exhibited enhanced insulation characteristics. This technique has mostly been replaced by applying rotating wire brushes. However, teasels are still employed, where a mild raising achievement is required [60].
- Cropping: Cropping finishing is used to cut protrude surface hair from a fabric to provide a smooth look which is usually employed on woolen merchandise where the elimination of surface hair by a singeing method is not achievable. The cropping process is performed by a spiral cutter rotating against a stationary blade cutting off any matter jutting from the fabric surface. By increasing and reducing the height of the cutting bed, the cutting depth could be changed, while the accumulated cut pile is removed by a strong suction process [61].
- Compressive shrinkage: It is defined as the mechanical shrinking of warp fibers leading to shrinkage of textiles on washing is decreased to the desired levels by the creation and processing stresses on the clothing [62].
- Heat setting: The major target of heat setting finishing is to guarantee that fabric does not shrink upon usage. This is mainly significant for applications, such as drive and time belts, where stretches can result in severe troubles. It is significant to test the reasons behind this failure in stability so that a complete recognition can be achieved on the effects that both heat and mechanical forces have on the stabilization of textile material [63].
- Chemical processes: These involve the application of chemicals to the cloth to introduce a variety of functions, such as water-repellent or flame-retardant properties, or to change the handle of a fabric. Chemical finishing is usually used in either an aqueous solution or emulsion type. It can be employed via various methods, the main method is the padding mangle which is usually followed by drying to eliminate the water from the fabric and make it ready for fixation of the finishing processes. This normally occurs by the baking process, where the textile is exposed to heating for a short time, to enable the applied finishing chemicals to afford a more durable finish [64].

- Durable flame-retardant processing

**Figure 11:**
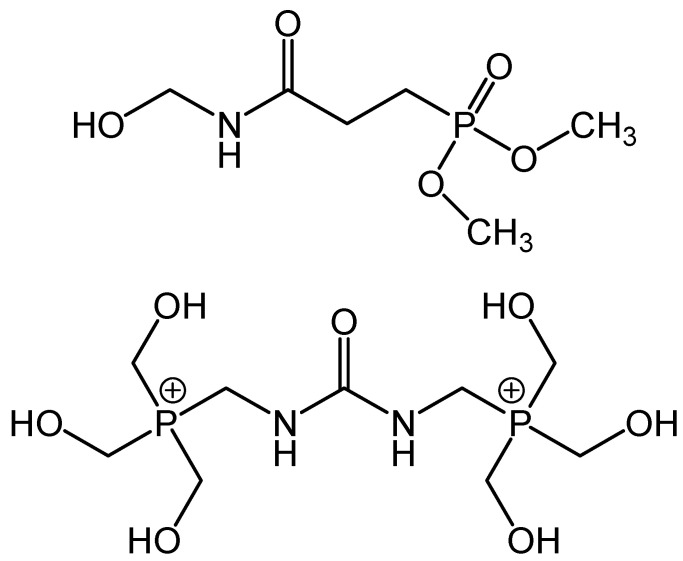
Chemical structures of Pyrovatex (**top**) and Proban (**bottom**).

Flame-retardant fabrics have been produced from various textile fibers. The major fibers used in this area is cotton. There are two key flame-retardant treatments are well-liked. These are the Proban and Pyrovatex treatment finishing processes (Figure 11). The Proban method uses phosphorus-containing materials based on tetrakis(hydroxymethyl)phosphonium chloride. This can be interacted with urea followed by padding into cotton fabric and partially dried to a residual moisture of 12%. The partially wet fabric is then subjected to ammonia gas and oxidized by hydrogen peroxide [65]. The fabric is then rinsed under tap water to remove the excess amounts of the phosphorus-containing material and hydrogen peroxide, dried in an oven at 45 °C, and finally washed with a fabric softener to reduce the harshness imparted to the fabric during the flame-retardant treatment process. The Proban method is fairly simple as it generates an insoluble polymer within the fiber gaps and voids of a cotton yarn. There is no actual chemical bonding to the cellulose polymer strains but the insoluble Proban is immobilized mechanically within the fibers and yarns. Thus, textile materials treated with Proban method have, to some level, harsh handle and accordingly, softeners are applied during the production process. The Pyrovatex technique is strongly correlated to cross-linked resins employed in textile finishing processes and is in fact always used with a cross-linked resin to create a chemical bonding with the hydroxyl functional group of the cellulose fibers. Pyrovatex is applied by padding, drying at 120 °C, curing at 160 °C for 3 min, washing in a dilute aqueous solution of sodium carbonate, washing in water, and finally drying with stentering to width. Compared to the Proban technique, Pyrovatex affords more durable fabrics due to bond formation between the flame retardant material and the fabric cellulose. Furthermore, due to the use of crosslinked resin, the finished fabric has high-quality dimensional stability and crease-recovery which is preferred for curtains. However, the application of Pyrovatex leads to the loss of tear strength, which usually takes place with all crosslinking agents [66].

- Water-repellent finishing

The early superhydrophobic finishing processes were dependent on applying a mixture of waxes. These were suitable to sail and protective clothing, but troubles were encountered upon cleaning. It was observed that the heavy metallic soaps possess superhydrophobic characteristics, and consequently the primary effort at the manufacture of a durable water-repellent finishing was to employ the chromium salt of a fatty acid to be applied on cotton followed by baking. The most recent water-repellent finishing processes include the application of fluorocarbons which are essentially an ester of perfluorinated hexanol with polyacrylic acid [67,68].

- Antistatic finishing

When dissimilar materials are rubbed together, a static charge is produced followed by separation of these charges leading to the formation of one positively charged material and the other one is negatively charged depending on the nature of the two materials. Under ambient conditions, cotton fibers possess very good antistatic properties due to the high moisture of 8% in cotton because it introduces the fiber with adequate conductivity to dissipate any charges that might be accumulated. One of the most remarkable developments of antistatic treatments has been the production of the durable antistatic finishing processes, such as the commercial Permalose antistatic agent, which is a series of treatments using block copolymer consisting of ethylene oxide and a polyester [69].

- Antimicrobial and antifungal finishing

**Figure 12:**
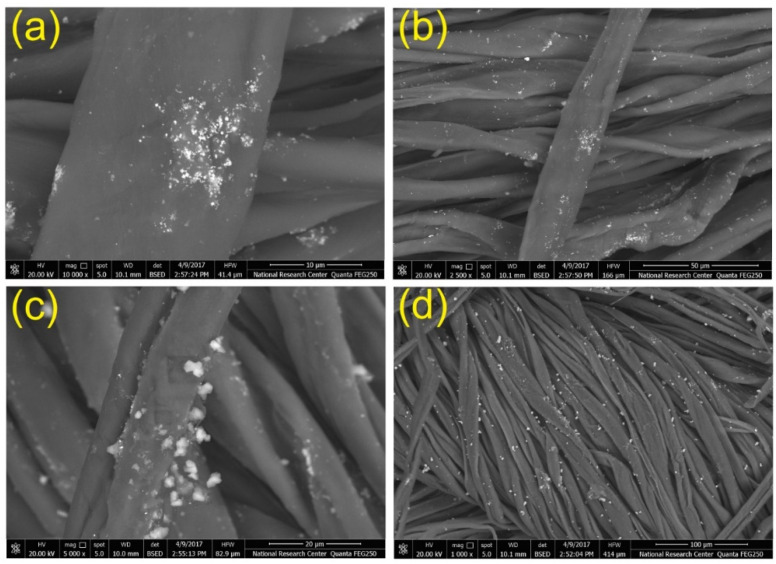
SEM images of antimicrobial plasma-pretreated cotton fibers coated with polyaniline (**a**,**b**), and polyaniline/silver nanoparticles composite (**c**,**d**) [70]. “Reprinted with permission from Springer Nature [70]”.

Antimicrobial and antifungal finishing (Figure 12) processes are very important in certain textiles for a variety of medical hygienic applications to avoid infections [70]. Antimicrobial finishing is largely used in garments such as mattress ticking, blankets and pillows that are being handled continuously by people as in hotels, hospitals, asylums and student hostels. For example, both zinc dimethyldithiocarbamate and copper 8-hydroxyquinolate have been used as microbicidal chemicals [71,72,73].

- Coloration of technical textiles

The dyes used for high-performance textiles can be chosen from an extremely broad range of synthetic or natural organic dyestuffs derived from aromatic materials of conjugated molecular structures. These conjugated systems have the capability to absorb particular wavelengths of the visible light, so that the completing light is scattered by the dyed high-performance fabric is perceived as colored. The dyestuff chemical structure has to enclose a chromophore and a functional group responsible for the color, such as nitro, hydrazon, and azo groups. To become a practical colorant, however, the dye molecular structure should enclose other functional groups such as amino, hydroxyl, sulphonic or carboxylic groups known as auxochromes. These auxochromes are responsible for modification or strengthening color, increase the dye solubility in water, and mainly assist attaching the dye to the fiber via either chemical or physical bonding. Elevated substantivity supports the degree of dye exhaustion onto the fibers from the dye-bath to provide colored technical textiles, and consequently, affords high color fastness properties such as rubbing, washing, sublimation, perspiration, and light [74,75].

**Figure 13:**
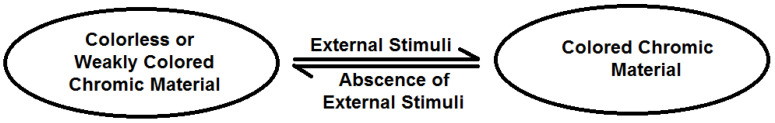
Behaviour of chromic materials or dyes.

**Figure 14:**
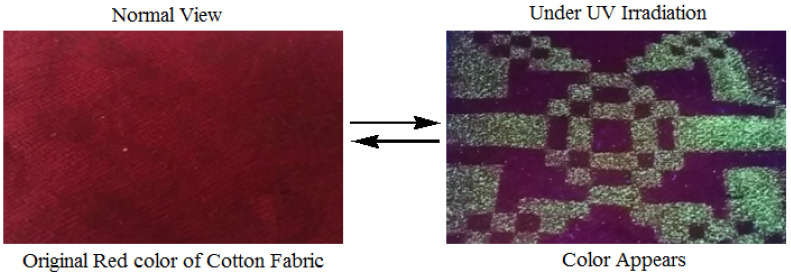
Photochromic effect of screen-printed cotton before and after irradiation with ultraviolet [77]. “Reprinted with permission from Elsevier [77]”.

**Figure 15:**
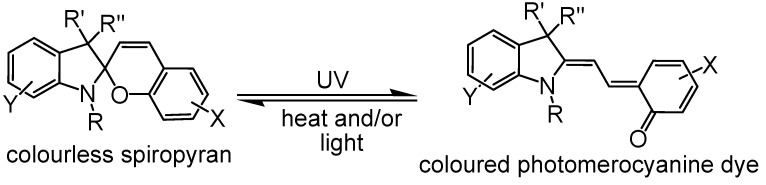
Photochromism of spiropyrans; X, Y = H, nitro, halogen, … etc.; R = alkyl; R′, R″ = alkyl.

Identification of dyes according to their application or their chemical structure are major approaches for classifying dyestuffs. Color is the most visible instant feature and the major aesthetic concern in textiles. Conventional dyestuff offers textile merchandise with expected, stable and permanent color. On the other hand, chromic dyestuff displays distinctive color transformations upon exposure to external stimuli (Figure 13 and Figure 14). Such color changes are usually controllable and reversible. For instance, photochromic dyes (Figure 15) change their color when subjected to ultraviolet radiation and return to their original color state when the light source is removed [76,77]. Thermochromic dyes can also change color in upon exposure to different temperature values [78]. Chromic textiles have the capability to change color presenting an opportunity to function as a form of flexible communication display. A chromic material is explored for specific functionality in technical smart clothing, also called chameleon textiles, to sense and respond to particular environmental stimulus. The most applied chromic materials in technical textiles are photochromic and thermochromic. Technical textiles are usually prepared by incorporating such smart materials by different including dyeing, printing, finishing, or direct incorporation during fibers production [79,80,81,82,83,84,85]. There is a variety of chromic behavior that depends on the type of chromic material (Table 1).

Coloration of technical textiles is performed either by dyeing to form uniform color or by printing to introduce a design or a pattern to a fabric. It is mainly anticipated for aesthetic motives, but also affords ready methods for recognizing the quality or fineness of materials [70,86]. For instance, the visibility of a fineness surgical suture at the implant position can be easily recognized by color. The high visibility of garments and camouflage coatings obviously afford the extreme ends of the coloration spectra for high-performance fabrics. Colorants can cover fiber yellowing and assist fiber protection against weathering, both aspects are of significance where the physical characteristics of the high-performance fabrics have to be maintained. High heat absorption is also raised whereas black garments are subjected to sunlight, a significant aspect for the packaging of agriculture products [87,88,89].

Coloration of technical textiles is sophisticated because of the huge variety of natural and synthetic fibers, yarns, and fabrics and the diverse nature of the end-use application. The ability to dye fibers, yarns and fabrics introduce a simple offer toward multicolored garments by weave or knit different colored yarns. In addition, the dyestuffs employed could be either water-soluble (or sparingly water-soluble) dyestuffs or water-insoluble pigments. The majority of dyestuffs are applied to dye textiles in an aqueous environment, although disperse-type dyes can also be applied via supercritical fluid carbon dioxide. On the other hand, pigments are either physically trapped inside the filaments during extrusion of polymers, or adhered to technical textiles by coating using an adhesive binder [90,91].

Dyeing is usually performed on textiles from which surface contaminants such as fiber lubricants, particulate dirt or natural coloring materials etc., are removed by the proper pre-treatments such as desizing, scouring, and bleaching. Various synthetic fibers do not usually necessitate chemical bleaching preceding to coloration since such fibers may be already whitened using a fluorescent brightener during the manufacturing process. The printing process can be performed mainly on high-performance textiles that may be in their natural state, bleached, whitened, or after dyeing [51,92,93,94,95,96,97].

- Conductive textiles

High-performance textiles can be considered as a growing research field especially for electroactive textiles (fabric, thread or yarn). The physicomechanical properties of textiles make them preferred solid state matrices for immobilizing a variety of substances. The advantages of electroactive textiles derived from their large surface area and high-quality mechanical and exploitation characteristics such as flexibility, conductivity, softness, strength, lightness, and capability to permeate gases. The large contact surface of textile fabrics usually allows efficient fit and flexibility. Research on sewing electroactive polymers into technical textiles is limited. Wearable conductive clothing opens new opportunities for developments in biomonitoring, rehabilitation, telemedicine, high-frequency shielding public telecontrol and teleassistance systems, static dissipation, wearable wireless communications, ergonomics and virtual-augmented reality. The electrically conductive technical textiles present a significant component in the recognition of electronic textiles. Furthermore, such electrical responsive properties can also be employed to incorporate chemical sensing capabilities for military purposes as protective clothes against hazardous biological and/or chemical environments [98,99].

A conductive polymer can be defined as a material able to respond to an applied electrical field by changing shape and/or dimensions. The applications of electroactive polymers have been mainly involved in medical, military and industry. Very promising progress in conductive materials science and technology, supports the recognition of conductive devices for electronic wearable textiles. In fact, there are different functions necessary for such interactive designs, including sensors, actuators, computation systems, and power generation/storage, which can currently be exerted by devices based on conductive fibers. Conductive fibers offer several advantages, such as light weight, significant elasticity, flexibility, low-cost and easy to process. They can be produced under different forms, printed, sewn, or knitted fabrics, or even woven in fibrous shapes directly into textile architectures. In addition to electroactive polymers, metal traces, such as gold, copper, silver, titanium and nickel, can be printed or deposited directly onto the fabric surface employing certain approaches, such as screen printing and vacuum deposition and sputtering [100,101].

## 4. Textile-Reinforced Composites

Textile-reinforced composites are a division of the broad category of engineering materials which is generally divided into four categories, including ceramics, polymers, metals and composites. An accurate meaning of composites is not easy to accomplish since the other three categories of homogeneous materials are sometimes heterogeneous at the submicron size. Composites are distinguished by being multiphase substrates inside which the phase distribution and geometry was intentionally modified to optimize one or more property. This is obviously a suitable description for textile-reinforced composites which is characterized by one phase, known as a matrix, reinforced by a fibrous strengthening in the form of a fabric. There are a variety of well-known fibrous blends available for textile-reinforced composites with a broad range of materials [102,103].

The total variety of potential composites is huge. In the case of reinforcements, we have to incorporate S-glass, R-glass, boron, carbon, ceramic and aramid fibers, and identified that the reinforcements can occur in the shape of short, long, plate, disk, sphere or ellipsoid fibers. Different reinforced materials have comprised a broad range of polymers, metals, and ceramics. Processing techniques are such as hand lay-up, autoclave, resin-transfer molding, powder-metallurgy methods for metals, injection molding for polymers, squeeze casting and chemical vapor permeation. The market for composites can be classified into two classes, including reinforced plastics derived from short fiber E-glass-reinforced unsaturated polyester resins (reported for more than 95% of market capacity); and advanced composites derived from advanced fibers, such as carbon, boron, aramid, or silicon carbide, or other advanced matrices, such as a high-temperature polymer matrix, and a metallic or a ceramic matrix, or advanced processing methods [104,105]. 

Textile-reinforced composite materials have been in use for engineering purposes for several years for comparatively cheap applications, such as woven glass-reinforced polymer hulls for mine sweepers. Textile reinforcement can replace different metal technologies. Textile-reinforced composite materials show potential for decreased production costs and improved processing, or in some cases, enhanced mechanical properties. Therefore, it is also competitive with comparatively mature composite technology that employs further conventional techniques of autoclave and prepregging production. There are different types of textile reinforcements, including weave, braid, knit and stitch. Woven textile reinforcement for polymer matrices is currently considered to be mature end-use purposes. The knitted glass textile materials drawn above the mold and injected by a resin employing the resin transfer molding processing method, has been employed to produce door constituents for a helicopter with the anticipation to replace the existing production method with the epoxy resin prepreg material and the autoclave processing of carbon fiber. Numerous textile processing methods are liable to be merged for some end-use purposes, such as the combination of braid and knit that can be applied to manufacture an I-shaped architecture. For structural end-use purpose, the characteristics which are resistant to damaging and/or cracks growth, strength and stiffness [106,107].

## 5. High-Performance Applications

The market size and growth of every key application area for high-performance textiles has been identified by Techtextil. Ecological technical textiles were recognized as a potentially significant growth division of technical textiles but are not calculated in the entire consumption since they have been identified under other divisions such as industrial textiles for production of filters and oil spill treatments; and Geo-Tech for geomembranes for toxic wastes and erosion protective clothing, etc. High-performance fabrics can be divided into many groups, depending on their functional characteristics. Such a categorization scheme was introduced by Techtextil, Messe Frankfurt Exhibition GmbH [4,9,23,108].

### 5.1. Transportation Including Automobiles, Shipping, Railways and Aerospace (Mobil-Tech or Mobil-Tex)

Mobil-Tech textiles are generally employed in the manufacturing of railways, automobiles, heavy trucks, ships, aircraft, and spacecraft. They are used as truck and car trunk covers, airbags, parachutes, timing belts, boats, engine noise insulation, higher end tires, seat covers, safety belts, air filters, and air balloons. Transport applications constitute the biggest single end-user area of high-performance textiles. Technical transportation textile goods range from rugs, seats, tires, seat and timing belts and safety air bags, to reinforced composites for automotive and aircrafts, including wings and engine machinery contents. The automotive industrial applications represent the highest commercialized section of all transportation high-performance textiles. The growth rate of novel other end-user applications such as air-bags and reinforced composite materials is expected to continuously exceed above averages by a large margin in coming years. Increasing the complexity of product qualifications and end-use of textile materials has resulted in the adoption of lightweight, low-cost, stronger, more durable and more accurately engineered yarns, woven and knitted garments and nonwoven. For example, the reduced weight per tire of textile reinforced cords in modern radial manufacturing. Internal technical textiles in automobiles also make use of lighter and inexpensive nonwoven. Reinforced hoses and belts are now able to last for a longer vehicle lifetime, replacing much of the huge and continuing textile goods from the market. The automotive industries have become increasingly worldwide players in an extremely competitive market. The providers of technical textiles to such a market are already predominated by a small number of large companies in each product area [109].

### 5.2. Medical and Hygiene (Med-Tech or Med-Tex)

Textiles that find hygiene and medical applications are termed high-performance medical textiles. The main applications include surgical gowns, drapes, wound care products, diapers, sutures, sanitary napkins and sterile packaging. The most well-known applications of high-performance textiles are for hygienic products, such as sanitary products, diapers and wipes. Manufacturers currently seek to develop both medical and hygiene textiles further by adding value toward more complicated products. Nonwoven predominate in such applications which are reported for more than 23% of all non-woven end-uses, the major sector of any of the 12 main high-performance textiles markets. Fears have been addressed at the increasing of disposable goods and the load that they put upon landfill and other waste disposal techniques. Attempts have been performed to establish biodegradable products for such end-uses but expenses are still high. An additional area of medical and hygiene sophisticated textile market is medical and surgical merchandise, such as operational gowns and drapes, arteries, sterilization packing, artificial ligaments, dressings, veins, skin replacement, hollow fibers for dialysis equipment, sutures, orthopedic pads etc. [110,111,112].

### 5.3. Household Textiles and Floor-Coverings (Home-Tech or Home-Tex)

High-performance home textiles (Home-Tech) are used for internal decoration, furniture, carpeting, floor and wall cover, sun shielding, and fire retardant. These are employed in the large market particularly as a fire retardant in buildings, ships, caravans, transportation, etc. Fire retardant characteristics are usually gained either by using inbuilt fire retardant such as modacryl fibers or by applying a coating containing fire retardant components such as phosphorus-based Pyrovatex materials. Hom-Tech textiles are by far the highest area of use compared to the other major 11 areas employing nonwoven and composite reinforcement materials. More than 35% of the entire weight of fibers and textiles in this class, are in the area of household furnishing and garments particularly in loose fibers in wadding. Hollow fibers of high-quality insulation are largely used in bed and sleep bags. Additional classes of fiber have been increasingly employed to replace foams in furniture packaging due to fear over flames and health hazards formed by those materials. Woven fabrics are still employed to a considerable level as furniture and carpet-baking products and curtain header tapes [113,114].

### 5.4. Agriculture, Aquaculture, Horticulture and Forestry (Agro-Tech or Agro-Tex)

Agro-Tech textiles are characterized by elongation, stiffness, biodegradation, and strength as well as protection against toxic environment and sunlight. Textiles employed in agriculture such as erosion and crop protection, layer separation in fields, sunlight screening, wind shield packaging for storing grass, and anti-birds nets. Light-weight spun-bonded fleeces are nowadays used for a variety of products, such as shading, weed suppression and thermal insulator. Heavyweight non-woven, woven and knitted textiles have been employed in hail and wind shelters. Capillary non-woven fibrous architectures have been used in horticulture to distribute moisture on rising plant. The mass storing and transportation of fertilizers and agriculture crop has been increasingly undertaken employing woven polypropylene-based flexible vessels to replace jute, paper or plastic packs. Agriculture is also an essential consumer of high-performance textile merchandise from other end-user segments such as Geo-Tech textiles for land reclamation and drainage, Pro-Tech garments for workers who must handle sprays and hazardous tools, and Mobil-Tech textiles for tractors and lorries, conveyor belts, hoses, filtration systems and reinforced composites for building silos, tanks and pipes. Fish farms have been a growing industry employing specialized net systems and other technical textile products such as lightweight strong lines and nets produced from dyneema and spectra fibers [115,116].

### 5.5. Filtration, Conveying, Cleaning and Other Industrial Uses (Indu-Tech or Indu-Tex)

In general, those are strongly woven textile materials characterized by high tenacity polyester and/or nylon yarns. They have been employed for electrical, mechanical and chemical engineering applications, such as plasma screens, transportations, lifting and grinding machinery systems, filtration, insulators, sound proofing, roller covering, and fuel cells. Separating solids from liquids or gases is a vital division of many industrial processes. The field of filtration systems can be recognized as the capturing of particles in the range of several millimeters down to the molecular level. There are requirements and standards that must be accomplished to produce a certain filter. Technical textiles have been typically used in filtration systems for the separation and cleaning of gases, fluids and effluents. Thus, textile filters are highly rising up in the worldwide market upon increasing manufacturing and environmental demands. There are five main categories of filtration system that can be best divided into liquid-liquid, solid-solid, solid-liquid, gas-gas and solid-gas filters. The textile permeability can be identified as the permission of such fabrics to allow the flow of certain molecules through it which necessitates engineering precise properties into a functional fabric depending on the desired product, and the features of the solids being filtered. Selecting and fabricating textile materials of certain properties is critical to the efficiency of a particular filter and its processing ability with a specified slurry composition to prevent any probable problem, such as filter plugging which results in low durability owing to the accumulation of the solid particles being separated [117].

### 5.6. Building and Constructions (Build-Tech or Build-Tex)

These are employed in construction including concrete reinforcement, frontispiece, interior architectures, sewer and pipes, linings, noise and heat insulation, fire and water proof, air conditioning, house-wrap, wall-reinforcement, aesthetic, and sun protective products. Impressive examples of Build-Tech are found in stadia, theaters, airports and hotels. Build-Tech textiles have been used in numerous ways in the assembly of buildings, dams, bridges, tunnels and roads. Temporary constructions, such as awnings, marquees and tents, have been used in a variety of applications due to their lightweight, strong, fire-retardant, rot-resistant, sunlight protection, and weather-proof characteristics. Nonwoven glass and polyester garments are already broadly employed in roofing, permeable membranes to stop moisture diffusion of walls, and insulation in building and machinery systems. Double walled spacer textiles can be filled with appropriate materials to afford sound and/or thermal insulators. Glass-reinforced composites involve septic tanks, wall panels and sanitary fittings. Polypropylene, glass and acrylic textiles have been employed to stop cracking of concrete and other construction defects. Carbon fibers are attracting attention as a potential reinforcement for earthquake-prone constructions although cost remains a significant restraint upon its more extensive uses. Textiles are also broadly in use in different construction processes, such as safety nets, lift and tension ropes, and flexible shutters for curing concrete. The possible applications of high-performance textiles for construction purposes are nearly unlimited [118].

### 5.7. Packaging (Pack-Tech or Pack-Tex)

Pack-Tech textiles are used for packaging, storing silos, containers, tents, and clothes covering.

Significant applications of textiles involve the development of sacks and bags, conventionally from jute, flax and cotton but currently from poly(propylene). The strengthened high-performance textiles emerged with modern substances handling techniques, have allowed the innovation of the more efficient handling, storage and distribution of diverse granular and powdered products varying from sand, cement, flour, sugar and fertilizer to pigments and dyestuffs. A growing sector of the packaging market employs light-weight knitted and non-woven materials for diverse wrapping and protective applications, particularly in foodstuff industry such as tea and coffee bags employing wet-laid nonwoven fabrics. Meat, vegetable and fruits are commonly packed using nonwoven introduced to absorb liquids. Other vegetable and fruits goods are provided in knitted net packing [119].

### 5.8. Sports and Leisure (Sport-Tech or Spor-Tex)

These were designed for shoes, cycling, summer and winter sports, angling, sail and fly sports, climbing, and sport equipment. After exclusion of applications of textiles in high-performance clothing and footwear, there are various applications of high-performance textiles in leisure and sport. Such uses are diverse and vary from synthetic grass employed in textile surface to carbon fiber reinforced composites for fishing rods, racquet and cycle frame as well as golf club. Further applications include garments for balloons, parachutes, paragliders and sailcloths [120].

### 5.9. Geotextiles and Civil Engineering (Geo-Tech or Geo-Tex)

Geotextiles are used in supporting of embankments, bridges, and drainage systems, while permeable Geo-Tech has been employed for soil reinforcement, erosion control, and filters. Revegetation of such textiles supported embankments or banks of water streams can also be promoted by applying the proper materials. Geotextiles are characterized by superior strength, durable, low moisture absorption and thickness. Geotextiles are typically non-woven and woven garments. However, man-made fibers such as polypropylene, glass and acrylic textiles are employed to avoid cracking of concrete and other building products. An increasing number of applications in the area of civil engineering, such as stabilizers, filters and other reinforcements are anticipated to increase demands for industrial textiles. Nonwoven constitute up to 80% of geotextile products. Modern interest is in composite-based textiles that merge the benefits of different textile structures, such as knitted, woven and non-woven as well as membranes [121].

### 5.10. Personel Safety and Protection (Pro-Tech or Pro-Tex)

Technical protecting clothing is commonly designed to improve workers’ safety according to requirements and regulations, to fulfill by technical textiles, described by organizations around the world such as American Society for Testing and Materials (ASTM) and International Organization for standardization (ISO). Technical protective clothing has been used for additional protection values against hazards such as high-temperature insulation particularly for fire-fighters, radiation in nuclear reactors, electric arc flash discharge, molten metal impacts, metal sparks in welding, highly acid/alkaline environments, bullet impact, and astronaut’s kit. The protection functional textiles, involves safety against stabs, explosives, fire, foul weathering, cuts, temperature (hot or cold), high voltage, abrasion, and dangerous dust and tiny particles, as well as chemical, biological and nuclear hazards. Similar to humans, sensitive equipment and processes also require protection. Therefore, dirt free room clothing is a vital condition for various industries such as electronics and pharmaceuticals [122,123].

### 5.11. Technical Components of Footwear and Clothing (Cloth-Tech or Cloth-Tex) 

Cloth-Tech is high-performance textiles for clothing purposes particularly for smooth finishing procedures where the cloth is treated under pressure and high temperature. This class of technical textiles involves yarns, fibers and textiles employed as technical elements in the production of clothes, such as waddings, interlinings, sewing threads and insulators. Some of the most recent and highly complicated advances have seen the inclusion of temperature phase changing materials into those insulating merchandise to offer an extra level of control and resistant character to sudden extreme changes of hot or cold temperature [124].

### 5.12. Environmental Protection (Oeko-Tech or Oeko-Tex (Eco-Tex))

Technical textiles have been used for safety purposes and environmental protection, such as air and water filtration systems, erosion defenders, oil spill management, floor sealing, and waste handling. Oeko-Tech textiles have been used for environmental protection. It is not a well-recognized class yet, though it has been overlapped with many other sectors of high-performance textiles, such as Indu-Tech textiles in filters, Geo-Tech in erosion protection and sealing of toxic wastes, and Agro-Tech in reducing water loss from soil and decreasing the necessity of herbicides by affording mulch to plants. Enhanced recyclability of technical textiles is becoming a significant concern not only for packaging but also for products such as automobiles. The current use of non-biodegradable thermoplastic composites has been the main reason for the reduced recyclability of the industrial technical textile products. Thus, there has been a substantial interest in developing recyclable biodegradable thermoplastic composites [125].

## 6. Sustainability and Ecological Aspects

The textile industries can be considered as one of the major reasons for the general decline in global environmental harm, pollution, and depletion of resources. Production, coloration, finishing, and textiles circulation of fibers, yarns or fabrics are performed with the support of massive, complex, costly machine systems and a variety of chemical materials. Hence, there are numerous possibilities for materials such as textile components or reagents employed for processing, to escape these machine systems leading to environmental pollution. Furthermore, efforts to manufacture all desirable merchandise lead to spreading impurities into air, water and soil, in addition to the undesired noise and ugliness of visible view [126].

### 6.1. Air Pollution

Air pollution from textile industries influences humans, machinery systems and final goods. There are growing health harms due to the textile industries, such as tuberculosis, byssinosis and asthma. Air pollution may also occur upon using textiles after production and during the end-use by consumer. For home textile furnishings, various pollutants are due to construction substances, but furniture, rugs, clothing, and wood or fabric furnishes most likely give rise to further customer criticism. This can be attributed to the existence of formaldehyde or other volatile organic materials. Secondary emissions from floor covers include harmful materials such as formaldehyde released, for instance from back coatings. However, technical textiles can play a vital task in reducing air pollution using filter fabrics able to remove particles of different particle sizes. The tiny pores in a fabric are perfect to prevent the diffusion of contaminants while permit air-flow to occur. Filtration fabrics, certainly, outline a main category of high-performance textiles [127].

### 6.2. Water Pollution

Water pollution is highly susceptible compared to other types of pollution systems to be connected with textile industries by public, mostly for the reason that, when it takes place, proof of its presence in the form of bio-accumulative organic materials, mutagenic chemicals, coloring dyes or pigments from printing and/or dyeing or detergent from washing and/or scouring is visually obvious [55,128,129]. Textile industry pollution can lead to abnormal pH levels of water streams. Pollution from textiles wet processes has reached shocking degrees, and several studies have been developed to decrease such water consumption and contamination by modifying industrial processing techniques, reducing the concentration of wastes, using optimal amounts of colorants or auxiliaries/chemicals of an eco-friendly nature, and by performing an appropriate restorative treatment: applying less water in industrial processes, decreasing the number of stages in bleaching, and chemicals/auxiliaries recovery from water streams. Many finishing processes can create pollutant byproducts; for example, sizing materials and starch are often considered to be the highest reasons for pollution. Other finishing pollutant byproducts are such as flame-retardant, softeners, antistatic, stain-resistant, water-proof, and oil-repellent. Loss of lubricants or spinning oil from machines can lead to an unintended discharge of harmful materials, and spillage of fuels from vehicles can also happen. Such pollutants have toxic effects on aquatic organisms or the improvement of species, such as algae which eliminate oxygen from water affecting aquatic organisms. Furthermore, aquatic organisms can sometimes survive when ingest such hazardous pollutants to be transmitted up the food chain to influence human beings. One of the most significant applications of Geo-Tech textiles is to decrease pollution. Geo-Tech membrane fabrics have been used on the coasts of water streams to prevent the widespread release of pollutants from different sources, such as industrial wastes and oil spills. This can be accomplished by applying ditch lining, landfill lining and stabilization membrane fabrics onto vegetation banks to prevent losing precious topsoil and stop the motion of soil containing pesticides or other dangerous substances into water resources [108,130,131].

### 6.3. Soil Pollution

Fibers or chemicals can be harmful if their degradation under the effect of air, water or sunlight generates toxic agents. Examples demonstrating the problem involve a variety of toxic degradation products from nylon, polyester or other polymeric materials which have been discarded in water streams and find their way to landfill locations. Attempts taken to make them biodegradable involve the application of starch as a resource of microbial nutrition or the integration of a material can be decayed by ultraviolet (UV) irradiation, both approaches simplify the disappearance of waste substances. Regrettably, UV decomposition is only successful until the polymers are buried, from which, such polymers can find their way to water supplies, acting as pollutants similarly as if they had been thrown away directly into water supplies at first. Again, the important role of high-performance textiles in the form of barriers to this contaminant transfer is vital [127].

### 6.4. Noise and Visual Pollution

Noise pollution arises in, for example, twist, spin and weave processes. Unpleasantly, high noise may also occur from transportation systems or other equipment employed in loading, shipping or handling in textile industries. There are several effects that may arise from noise pollution, the most apparent effect being hearing loss and deafness. Other effects from high noise levels are psychological problems such as frustration, carelessness, withdrawal or sullenness. Nonetheless, technical textiles can serve in controlling the effects of noise such as common use of acoustic absorbent materials to decrease the unpleasant effect of high sound pollution. Paper documents and packaging, or plastic sheets employed to wrap textiles usually find their way into landfill locations or spread around to offend the eyewitnesses. Technical textiles, again, are helpful in reducing the crisis by applying their aesthetic character [132,133].

### 6.5. Reduction of Environmental Harm

Both renewable and non-renewable sources similarly are at threat from the desires of the textile industry. There are a variety of well-known resources, such as oil and petroleum products for the production of printing colorants, thickeners and binders, iron and other metals for equipment or colorants, water for the industrialized processes, trees for the manufacture of fibers. Eco-friendly textile green processing is introduced as a worldwide challenge. The harm reduction for ecological protection can be divided mainly into four categories including recycling toward resource depletion, using environmentally friendly fibers or other supplies, and enhancement of techniques employed to eliminate pollution after it has been generated. Technical textiles are often used in combination with other substances, such as coating and hardening oil, or as elements of fiber-reinforced composite. These products could be hard or not possible to degrade suitably into their original ingredients, or such end-products may degrade successfully to their harmful origin components. Recycling reduces wastes, water consumption, energy and chemical costs. Realistic solutions proposed the development of novel dry processing techniques instead of wet processing, dyeing using supercritical carbon dioxide, using plasma under vacuum or inkjet printing. Biotechnology has been suggested to decrease pollution toward a greener industry. An oxidation reactor with the ability to treat deeply contaminated water streams and decrease the use of both water and chemicals in textile finishing processes has been presented recently. Activated carbon is employed to decolorize polluted water from reactive dyestuffs, although additional efforts demonstrate that adsorption of dyestuffs onto peat has a comparable extracting aptitude, most probably due to surface changes and solution pH [125,134,135,136,137].

## 7. Future Trends

Technical textiles are one of the commercially growing industrial segments. The fast expansion of high-performance textiles and their applications have created many opportunities for different innovative applications. The market size of technical textiles is expected to surpass US$251.82 billion by 2027. Technical textiles were only used normally as wound-care goods, diapers, braces, prosthetics, wipes, breathing masks, bedclothes, ropes, and belts etc., but the technology has been upgraded toward many and diverse end-uses. The worldwide future of high-performance textiles can be considered as a broader economic trend than just the production and processing of textiles. Reducing ecological impacts from technical textiles on soil, air and water will be under more investigation. The future of technical textiles promises even stronger global competition, which will see producers seeking more applications, lower cost, and high-quality products.

## Figures and Tables

**Table 1:** Different chromic behaviors.

Chromic Phenomena	External Stimulus
Photochromism	Light
Thermochromism	Heat
Electrochromism	Electrical current
Halochromism	pH
Solavtochromism	Solvent polarity
Hygrochromism	Moisture
Mechanochromism	Mechanical deformation
Tribochromism	Mechanical friction
Piezochromism	Mechanical pressure
Chemochromism	Chemical agents such as explosives
Gasochromism	Gases
Carsolchromic	Electron beam
Vapochromism	Vapors of organic materials due to their polarity, pH, … etc.
Cathodochromism	Electron beam irradiation
Radiochromism	Ionizing radiation
Biochromism	Biological entity
Aggregachromism	Dimerisation/aggregation of chromophores
Crystallochromism	Variation in crystal structure of a chromophore
Magnetochromism	Magnetic field
Ionochromism	Ions
Chronochromism	Time
